# Occupational Exposure of Employees in a Multispecialty Hospital to Factors Causing Contact Dermatitis—A Questionnaire Study

**DOI:** 10.3390/medicina59122084

**Published:** 2023-11-27

**Authors:** Aneta Drozdowska, Piotr Drozdowski, Aleksander Jaworski, Anna Ryczek, Daniel Bula, Dawid Adamczyk, Jowita Adamczyk, Ada Łątkowska, Barbara Sławińska, Adam Reich

**Affiliations:** 1 High Medical School in Klodzko, 57-300 Kłodzko, Poland; aneta_drozdowska@wp.pl (A.D.); piotr_drozdowski@wp.pl (P.D.); 2 Specialist Medical Centre, 57-320 Polanica-Zdrój, Poland; 3 Este Derm, 33-100 Tarnow, Poland; ryczek.anna@wp.pl; 4 Oncological and Reconstructive Surgery Department, Maria Sklodowska-Curie National Research Institute of Oncology, Gliwice Branch, 44-102 Gliwice, Poland; daniel.bula@gmail.com; 5 Department of Radiology and Radiodiagnostics, Faculty of Medical Sciences in Zabrze, Medical University of Silesia in Katowice, 40-055 Katowice, Poland; da.adamczyk@gmail.com; 6 Ophthalmology Department, Faculty of Medical Sciences in Zabrze, Medical University of Silesia in Katowice, 40-055 Katowice, Poland; jowitkaa@gmail.com; 7 Faculty of Medicine, Wroclaw Medical University, 50-367 Wroclaw, Poland; adalatkowska01@gmail.com; 8 Faculty of Medical Sciences in Zabrze, Medical University of Silesia in Katowice, 40-055 Katowice, Poland; basiaaa97@gmail.com; 9 Adam Reich, Department of Dermatology, Institute of Medical Sciences, Medical College of Rzeszow University, 35-055 Rzeszow, Poland

**Keywords:** occupational dermatitis, contact dermatitis, health professionals, questionnaire study, dermatitis

## Abstract

*Background and Objectives***:** Allergic contact dermatitis (ACD) is a serious health and socio-economic problem. Accurate and reliable assessment of exposure to ACD factors in the work environment would increase quality of life and work of employees. The aim of this study was to assess the level of exposure of workers of a multidisciplinary hospital to the factors causing ACD. *Material and Methods*: The proprietary OSDES-16 questionnaire was used. The effectiveness of the OSDES-16 was confirmed statistically. The study included 230 employees of the medical center in Polanica Zdrój, divided into groups. *Results*: The differences in the overall assessment of exposure between the individual groups in the OSDES-16 scale were statistically insignificant (*p* > 0.05). There was no significant correlation between the current workplace and the level of exposure to ACD (*p* > 0.05). The level of exposure to ACD in the group of employees with work experience in the current position for more than 10 years was significantly higher than those working less than 6 years (*p* < 0.05). *Conclusions*: Nurses, midwives and paramedics are the occupational group most exposed to the development of contact allergy related to exposure to factors present in the work environment. The seniority of more than 10 years in the current position was linked with a higher level of occupational exposure.

## 1. Introduction

According to the data of the European Agency for Safety and Health at Work from 2008, dermatoses accounted for as much as 7% of the total percentage of diagnosed occupational diseases [1]. The most common of these is contact dermatitis (CD).

Contact allergy is a significant medical and social problem. According to the results of studies from different countries [2,3,4], the incidence of atopic eczema is estimated at 5–23%, allergic contact eczema at 17% and irritant eczema at 13%. Although Polish epidemiological data regarding the incidence of occupational skin diseases are underestimated, they undoubtedly contribute to the generation of significant absences from work and, consequently, higher costs for the employer. Allergic contact dermatitis (ACD) can arise from both irritating and allergenic factors. The available studies describe either selected allergens, allergies in selected occupational groups or selected disease symptoms or anatomical locations [5,6,7,8]. The gold standard of ACD diagnostics are the so-called “patch tests” [9]. Other additional tests, such as intradermal tests, “practical tests” or provocative oral tests, are also helpful. However, the professionals also have to keep in mind to precisely collect the medical history. Combining it with—even seemingly irrelevant—information about the patient’s daily habits, allows for an appropriate selection of the range of haptens in the patch tests performed. Collecting a comprehensive history requires knowledge about the most common allergenic factors in specific occupational groups, as well as about selected disease symptoms with their most frequent location. However, there are no studies that would correlate the incidence of given allergens with a given workplace or work environment. Therefore, the purpose of the following analysis was to investigate the prevalence of contact allergy to a wide spectrum of potential allergens among people working in various medical professions. Identification of allergens occurring in a given work environment can lead to optimization of the frequency of preventive examinations and limitation of exposure by the necessity to use personal protective equipment, and possibly also to partial removal of substances with a high allergenic potential from the immediate surroundings of specific hospital staff. The purposes of the following analysis were primarily to determine the frequency and clinical characteristics of contact allergy to selected allergens present in the work environment of a multidisciplinary hospital, taking into account various workplaces, among employees of the Medical Center in Polanica-Zdrój (SCM); to determine the relationship between the presence of contact allergy and demographic factors, other diseases and the job position; to select a group of employees presenting a greater risk of contact allergy, which would enable them to be provided with specialist dermatological care; and to compare the exposure of employee groups within hospital employees to risk factors for contact allergy. In order to achieve that, we used our proprietary scale of exposure to occupational skin diseases containing 16 questions (OSDES-16). The OSDES-16 is based on an occupational skin diseases’ exposure scale consisting of 49 questions (OSDES-49) (Table 1). The OSDES-16 is the shortened, yet statistically-proven equal clinical device to the OSDES-49. To test the reliability of the scale items of OSDES-49, the internal consistency method of the scale was used. It was assumed that the individual items of the scale should be correlated with the total score of at least 0.4 (Kleine’s criterion) and the Cronbach’s coefficient should be higher than 0.7 (Nunnally’s criterion). The OSDES-49 had a mean value (M) of 15.68 with a standard deviation (SD) of 7.45. The Cronbach’s alpha (α) coefficient for this scale was 0.891, and the average correlation coefficient (r) was 0.151. Following the established criteria for evaluating the reliability of the scale’s items and removing those that did not meet Kleine’s and Nunnally’s standards, the OSDES-16 containing only 16 items was derived. The mean value for the OSDES-16 in the group of employees from the SCM study was M = 5.89, with a standard deviation of SD = 4.76. The Cronbach’s alpha (α) coefficient for this reduced scale was 0.91, and the average correlation coefficient (r) was 0.397.

The assessment of the risk of developing occupational skin diseases, as determined by an OSDES-49, demonstrated a strong correlation with the assessment obtained using an OSDES-16. Spearman’s rank correlation coefficient (rho) was calculated as 0.85 (*p* < 0.001). The strong agreement observed between the evaluations of both scales, along with the feasibility of substituting the 49-item questionnaire with the 16-item version for screening purposes, was supported by a substantial Cohen’s kappa coefficient. This coefficient was measured at k = 0.639, and its 95% confidence interval was found to span from 0.556 to 0.722.

The statistical analysis revealed that the shorter version of the OSDES-49, which is OSDES-16, exhibited a sensitivity in identifying occupational exposure to contact allergy equivalent to that of the original, more extensive version, which contains 49 questions. Consequently, the OSDES-49 can be considered as a foundational tool, while the OSDES-16 can be viewed as a definitive and clinically practical screening instrument suitable for dermatologists. For a more elaborate description of the data regarding OSDES-49 and the comparison between OSDES-49 with OSDES-16, we encourage familiarization with our paper devoted entirely to those purposes [10].

## 2. Materials and Methods

**Figure 1:**
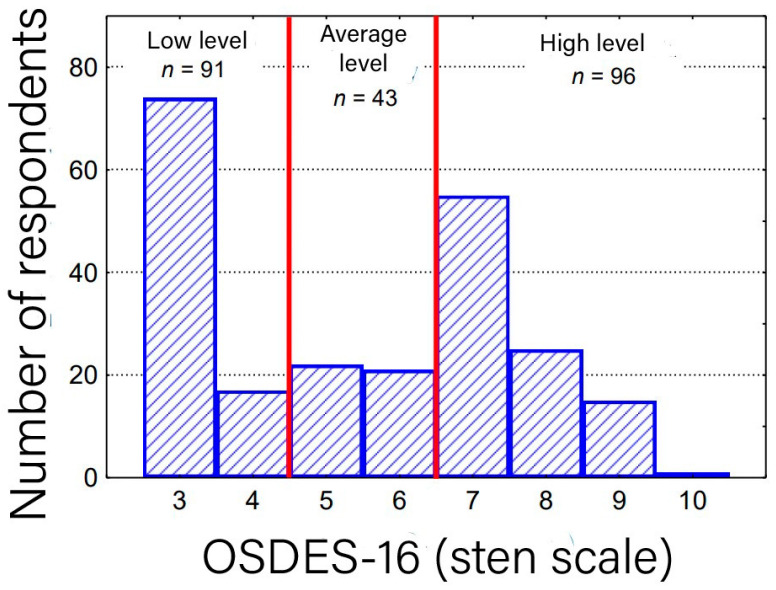
Histogram of the assessment of the level of occupational skin disease exposure among a group of 230 employees of SCM in Polanica Zdrój on a sten scale (OSDES-16).

The analysis included all employees of the Medical Center in Polanica Zdrój who underwent preliminary or periodic examinations in the field of Occupational Medicine Service. For the purposes of this study, an OSDES-16 was used (Table 2). For quantitative features (variables), mean values (M), standard deviations (SD), medians (Me), lower (Q1) and upper (Q3) quartiles and extreme values, the minimum (Min) and maximum (Max) were calculated. The qualitative features were presented in cross tables in the form of count (n) and proportion (%). The total score of the questionnaire, calculated on the basis of the adopted scale of exposure to occupational skin diseases, was transformed into a sten scale (Figure 1). Each individual test’s Person’s score (Xi) was converted to a standardized score with z using the expression z_(giving =) (x_i-M)/SD.

The current study included individuals who met the following inclusion criteria: employment at the SCM in Polanica-Zdrój, consent to participate in the study, reaching the age of 18, undergoing initial, periodic or follow-up examinations at the SCM with an occupational physician in the period from March to December 2017. Exclusion criteria for participation in the study were the occurrence of disseminated or generalized eczema (within the 4 weeks prior to the study), the occurrence of acute eczema, the occurrence of eczema in the test area, topical use of glucocorticosteroids or calcineurin inhibitors in the test area, use of systemic immunosuppression, use of phototherapy, persistent positive reactions to previously performed patch tests.

Ultimately, 230 people aged 23–85 were enrolled in the study, in accordance with the adopted criteria. In the studied group, the share of women was significantly greater than that of men (88.3% vs. 11.7%; *p* < 0.001). Since a strong positive linear correlation was observed between age, length of service and seniority in the current position, only seniority in the current position and the three groups of employees related to it were taken into account in the further analyzes, as follows: work experience in the current position less than 6 years (n = 74), work experience in the current position from 6 to 10 years (n = 87), work experience in the current position over 10 years (n = 69). Additionally, due to the occupied position and the associated exposure to contact allergy, SCM employees were divided into four groups: doctors (n = 21); nurses, midwives, rescuers, orderlies (n = 162); medical analysts, pharmacists (n = 17); others: technical staff, medical secretaries, administration staff (n = 30).

The statistical analysis was based on the “STATISTICA 13.0” software (TIBCO software, Kraków, Poland). In relation to quantitative variables, non-parametric U Mann–Whitney (for two groups) or Kruskal–Wallis (when there were more groups) tests of significance were performed. Pearson’s chi-square test or Fisher’s exact test were used to verify the hypotheses about the independence of two nominal or ordinal variables. The strength of the correlation between the two quantitative variables was determined by calculating the Pearson correlation coefficient r or the Spearman’s rank correlation coefficient rho. In all tests, the critical significance level was *p* < 0.05.

The guidelines of the Good Clinical Practice and the 2013 World Helsinki Declaration were used. All patients participating in the study were informed about the goals, methods and possible test results of the study. Before being included to the study, the patients were asked to sign their informed consent to participate in the study and give written consent to the processing of personal data. This research obtained the approval of the Bioethics Committee of the Wroclaw Medical University—decision no. KB—178/2017.

## 3. Results

**Figure 2:**
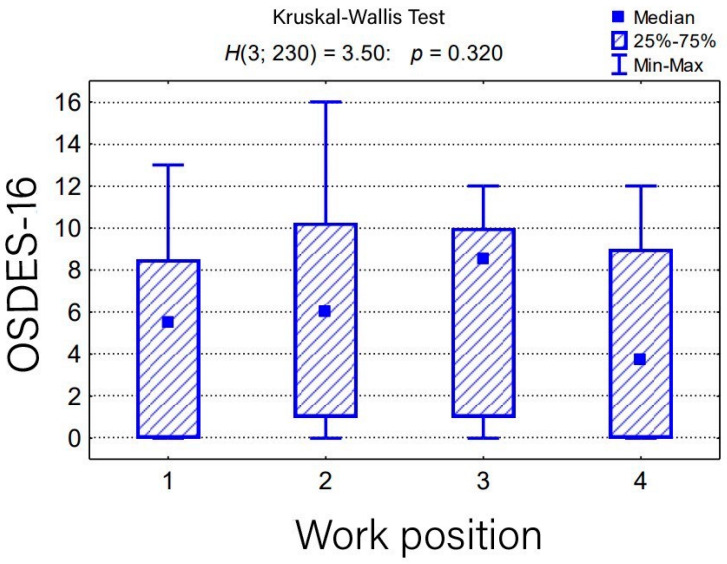
Overall assessment of the level of occupational skin disease exposure on the OSDES-16 scale in employee groups with different job positions, and the result of the Kruskal–Wallis test for significance, as well as post-hoc tests.

**Figure 3:**
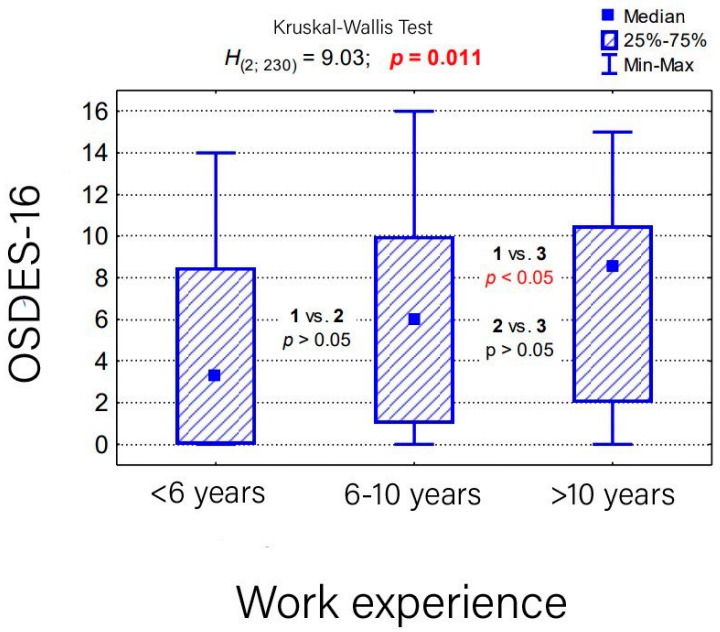
Overall assessment of the level of occupational skin disease exposure on the OSDES-16 scale in employee groups with different lengths of tenure in their current positions, as well as the results of the Kruskal–Wallis test for significance and post hoc tests.

Completed results of the survey are available in Table 3. The differences in the overall assessment of exposure between the individual groups (physicians vs. nurses vs. analytics vs. others) in the OSDES-16 scale were statistically insignificant (*p* > 0.05), except for the questions “Do the symptoms subside after days off work?” and “Do similar lesions occur in other employees in the same position?”, where affirmative answers were more common among the nurses (Table 4). Based on our assessment, women were found more exposed to occupational skin diseases, which results from their work positions (Table 5). There was no statistically significant relationship between the current workplace and the level of exposure to occupational skin diseases (*p* > 0.05) (Table 6) (Figure 2). The majority of the workers in group 4 (including mostly clerks) were classified as low-risk exposure and the nurses (group 2) were more frequently classified as high-risk exposure. The assessment of the level of exposure to occupational skin diseases in the group of employees with work experience in the current position for more than 10 years was significantly higher than the employees working in the current position for less than 6 years (*p* < 0.05) (Table 7) (Figure 3).

## 4. Discussion

The working environment of a multidisciplinary hospital with a surgical profile is heterogeneous—both due to the risk factors present there and the variety of work positions. The research descriptions available in the literature present either selected allergens or allergies in selected occupational groups, or selected disease symptoms or anatomical locations [5,6,7,8]. It seems reasonable to undertake research on the frequency of contact allergy among hospital employees, taking into account all professional groups and all disease symptoms, carried out on a large group of respondents. Allergic contact dermatitis is estimated to be the most common occupational disease of healthcare workers. According to the results of the study by Machovcowa et al. [11] on a group of 545 employees of this sector, ACD was responsible for 85% of diagnosed occupational diseases. The scale of the problem is therefore extremely important. ACD causes ailments that make it very difficult to work, and in some cases make it impossible. This leads to a deterioration in the quality of the employee’s work, and even the need to change the profession. On an individual scale, it contributes to financial problems, and globally, ACD negatively affects the entire economy. For this reason, it seems appropriate to investigate the degree of exposure to the causative agents of ACD in particular work environments. The authors of the current study attempted to link the demographic data with the job profile in a multidisciplinary hospital and medical history. The research was conducted in the form of a survey on a representative group of 230 employees. Due to the heterogeneity of workplaces, for the purposes of the statistical analysis, the group of employees was unified, guided by similar working conditions, scope of duties and the resulting homogeneous risk factors for skin diseases. The most common allergenic factors in the hospital environment include nickel, thiomersal, fragrances, rubber, cacophony and latex, phenyldienamine and formaldehyde [6]. Ibler et al. [5] described a group of 120 hospital workers in Denmark with hand eczema: 53% of the subjects tested positive for the above-mentioned allergens by patch tests. In turn, Turjanmaa [8] described the frequency of contact allergy to latex among 512 hospital employees. The result obtained with the “prick” tests was 2.9%. There are also studies available describing the prevalence of allergies to chlorhexidine, a commonly used disinfectant. Opstrup et al. [12], after examining a group of 8497 patients, determined its frequency as 1%. Disinfectants and personal protective equipment are not the only sources of allergens in the hospital environment. Metals commonly used in orthopedics are also responsible for causing CD [13]. It is estimated that protease-induced allergies are responsible for nearly 50% of surgical failures [14,15,16]. There were suggestions that it would be appropriate to introduce allergological interviews to routine diagnostics before orthopedic procedures with the use of implants, enriched with tests in the case of ambiguity. We strongly believe that our OSDES-16 scale could be useful in order to achieve this.

One of the most important assumptions of this study was to find an answer to the question of whether the factors to which hospital workers are exposed significantly increase the incidence of contact allergy, and if so, which occupational group is the most exposed to it. For this purpose, a proprietary questionnaire was used, in which personal data, job characteristics and medical history were taken into account. The OSDES-16 questionnaire can be proposed as a screening method that is easier to apply—both for the researcher and the respondent [10]. Based on the obtained results, nurses, midwives and paramedics are the occupational group most exposed to contact allergy related to exposure to factors present in the work environment among the employees of a multidisciplinary hospital. The above conclusion should be combined with the following premises: 1. The described professional group is characterized by the most frequent soaking of hands in the work environment, which negatively affects the tightness of the epidermal barrier, predisposing to increased skin penetration, e.g., by allergens. 2. In the described professional group, there is an increased exposure to detergents, the action of which also damages the skin’s protective barrier. 3. Nurses, midwives, paramedics and hospital attendants most often work in a 12 h system, which increases the length of exposure to harmful chemical factors. In the studied group, the share of women was significantly greater than that of men (203 vs. 27). This can be explained as follows: 1. Hospital workers are mostly women. 2. Women are more interested than men in testing the occurrence of allergies to allergens present in cosmetics due to their more frequent use. Overall, 100% of employees undergoing preventive examinations at the SCM company doctor underwent the survey there. For this reason, we believe that the study objectively illustrates the degree of exposure of individual professional groups working in a multidisciplinary hospital. It should also be borne in mind that the study did not involve employees employed on terms other than full-time employment, such as a mandate contract or contract. The decision not to include these employees in the study was due to the fact that they did not need to participate in periodic medical examinations carried out by an occupational medicine specialist. In the future, it would be useful to consider designing a study that would include all hospital staff, regardless of their form of employment. It should be noted that a large, multidisciplinary hospital with extensive administrative and technical facilities can be treated as a reliable model of a hospital. It seems that the results constitute a valuable reference point for subsequent studies in the described professional group and—in a broader sense—in the hospital environment. In addition, the obtained results will potentially enable the analysis of interdependencies between individual risk factors—examining possible synergistic, additive, potentiating or antagonistic mechanisms in the studied group of people. Nevertheless, this type of research goes beyond the assumptions of this paper and may constitute a possible starting point for further research.

## 5. Conclusions

Among the employees of a multidisciplinary hospital, nurses, midwives and paramedics are the occupational group most exposed to contact allergy related to exposure to factors present in the work environment. Disease symptoms are more common among women. Although the seniority of more than 10 years in the current position correlated with a higher level of occupational exposure, it did not contribute to an increased incidence of disease symptoms.

## Figures and Tables

**Table 1:** Scale of the level of exposure to occupational diseases of the skin consisting of 49 points (OSDES-49).

Item	Questions	Scoring
Do these phenomena occur in the workplace?		If “Yes”, circle the point
1.	Soaking hands	1
2.	Exposure to detergents	1
3.	Exposure to chemical disinfectants	1
4.	Exposure to chemotherapeutics	1
5.	Exposure to chemical reagents	1
6.	Exposure to other chemicals	1
	If yes please specify:	
7.	Wearing gloves (any type)?	1
8.	Wearing latex gloves?	1
9.	Wearing vinyl gloves?	1
10.	Wearing powdered gloves?	1
11.	Was personal protective equipment used?	1
12.	Was there a rotation of employees at work positions?	1
13.	Does the location of lesions correspond to occupational exposure?	1
14.	Do the symptoms subside after days off work?	1
15.	Do similar lesions occur in other employees in the same position in company?	1
If the skin symptoms occur, are they accompanied by:		
16.	Nasal discharge?	1
17.	Tearing, burning, redness of the eyes?	1
18.	Dyspnoea?	1
19.	Eczema?	1
20.	If yes, please specify the frequency of eczema occurrence	
	Occasionally	0.25
	Regularly, but less than once a month	0.5
	Regularly, at least once a week	0.75
	Regularly, at last once a week, but the lesions cease after a weekend or a break from work	1
Are there tendencies for the following skin lesions?		1
21.	Erythema?	1
22.	Exfoliation?	1
23.	Fissures?	1
24.	Erosions?	1
25.	Hyperkeratosis?	1
26.	Dryness of the skin/lichenification?	1
27.	Blisters?	1
28.	Ulcers?	1
29.	Burning and/or sore skin?	1
30.	Pruritus?	1
31.	Have you been diagnosed with atopic dermatitis?	1
32.	Have you been diagnosed with psoriasis?	1
33.	Have you been diagnosed with vitiligo?	1
34.	Are there any other skin diseases?	1
	If yes, please specify:	
35.	Are the lesions spreading outside the place of exposure?	1
36.	Is the onset of the lesions sudden?	1
37.	Do symptoms occur 24–72 h after exposure?	1
38.	Have you been diagnosed with bronchial asthma?	1
39.	Were patch tests performed?	1
40.	Have any other diseases been diagnosed?	1
	If yes, please specify:	1
41.	Have the following treatments been used?—systemic steroids, local steroids, calcineurin inhibitors, phototherapy	1
42.	Were any measures that help to restore the epidermal barrier used?	1
43.	Do symptoms occur after additional exposure to another irritant such as solar radiation?	1
	If yes, please specify after what period of time:	
44.	The most frequently affected area of the body	1, if hands
Are there tendencies for the following symptoms occurrence?		
45.	Dryness of skin	1
46.	Excessive activity of the sebaceous glands (Seborrhea)	1
47.	Excessive sweating	1
48.	Excessive pigmentation	1
49.	Frequency of burning and painfulness:	
	Occasionally	0.25
	Regularly, but less than once a month	0.5
	Regularly, at least once a week	0.75
	Regularly, at least once a week, but the lesions cease after a weekend or a break from work	1
Total score		

**Table 2:** OSDES-16: a scoring of exposure to occupational skin diseases.

Item	Questions	Scoring (Points Awarded for an Affirmative Answer)
1.	Do the symptoms subside after days off work?	1
2.	Do similar lesions occur in other employees in the same position in the company?	1
	If the skin symptoms occur are they accompanied by:	
3.	Nasal discharge	1
4.	Tearing, burning, redness of the eyes	1
5.	Eczema	1
6.	If yes, please specify the frequency of eczema occurrence	
	Occasionally	0.25
	Regularly, but less than once a month	0.5
	Regularly, at least once a week	0.75
	Regularly, at least once a week, but the lesions cease after a weekend or a break from work	1
7.	Erythema	1
8.	Dryness and/or lichenification	1
9.	Burning and/or sore skin	1
10.	Frequency of skin burning and/or painfulness	
	Occasionally	0.25
	Regularly, but less than once a month	0.5
	Regularly, at least once a week	0.75
	Regularly, at least once a week, but the lesions cease after a weekend or a break from work	1
11.	Pruritus	1
12.	Is the onset of the lesions sudden?	1
13.	Do symptoms occur 24–72 h after exposure?	1
14.	Have any measures that help to restore the epidermal barrier been used?	1
15.	Any area of the body affected	1
16.	Dryness of the skin	1
Total score:		

**Table 3:** Number of affirmative responses to individual survey questions.

Item	Questions	“Yes” n (%)
1.	Do the symptoms subside after days off work?	94 (40.9)
2.	Do similar lesions occur in other employees in the same position in company?	75 (32.6)
	If the skin symptoms occur are they accompanied by:	
3.	Nasal discharge	65 (28.3)
4.	Tearing, burning, redness of the eyes	98 (42.6)
5.	Eczema	138 (60.0)
6.	If yes, please specify the frequency of eczema occurrence	
	Occasionally	62 (27.0)
	Regularly, but less than once a month	19 (8.3)
	Regularly at least once a week	17 (7.4)
	Regularly at least once a week, but the lesions cease after a weekend or a break from work	40 (17.4)
7.	Erythema	62 (27.0)
8.	Dryness and/or lichenification	112 (48.7)
9.	Burning and/or sore skin	50 (21.7)
10.	Frequency of skin burning and/or painfulness	
	Occasionally	45 (19.6)
	Regularly, but less than once a month	18 (7.8)
	Regularly, at least once a week	15 (6.5)
	Regularly, at least once a week, but the lesions cease after a weekend or a break from work	31 (31.5)
11.	Pruritus	92 (40.0)
12.	Is the onset of the lesions sudden?	71 (30.9)
13.	Do symptoms occur 24–72 h after exposure?	57 (24.8)
14.	Have any measures that help to restore the epidermal barrier been used?	46 (20.0)
15.	Any area of the body affected	103 (44.8)
16.	Dryness of the skin	126 (54.8)
Total score:		

**Table 4:** Answers to the survey questions broken down by the position held.

Item	Position				*p* Test Score
	Physicians N = 21 (%)	Nurses N = 162 (%)	Analytics N = 17 (%)	Others N = 30 (%)	
1. Do the symptoms subside after days off work?	4 (19.0)	76 (46.9)	7 (41.2)	7 (23.3)	0.015
2. Do similar lesions occur in other employees in the same position in company?	3 (14.3)	66 (40.7)	3 (17.6)	3 (10.0)	<0.001
3. Nasal discharge	3 (14.3)	46 (28.4)	6 (35.3)	10 (33.3%)	0.42
4. Tearing, Burning, Redness of the eyes	4 (19.0)	72 (44.4)	9 (52.9)	13 (43.3)	0.125
5. Eczema occurs	11 (52.4)	100 (61.7)	12 (70.6)	15 (50.0)	-
6. Frequency of eczema’s occurrence					0.523
Does not occur	10 (47.6)	62 (38.3)	5 (29.4)	15 (50.0)	
Occasionally	5 (23.8)	41 (25.3)	7 (41.2)	9 (30.0)	
Regularly, but less than once a month	2 (9.5)	13 (8.0)	3 (17.6)	1 (3.3)	
Regularly, at least once a week	1 (4.8)	13 (8.0)	0 (0.0)	3 (10.0)	
Regularly, at least once a week, but the lesions cease after a weekend or break from work	3 (14.3)	33 (20.4)	2 (11.8)	2 (6.7)	
7. Erythema	8 (38.1)	41 (25.3)	5 (29.4)	8 (26.7)	0.659
8. Dryness, lichenification	9 (42.9)	83 (51.2)	8 (47.1)	12 (40.0)	0.652
9. Burning and/or sore skin	4 (19.0)	35 (21.6)	5 (29.4)	6 (20.0)	0.865
10. If any of the above occurs please specify, with what frequency?					0.875
Does not occur	10 (47.6)	85 (52.5)	8 (47.1)	18 (60.0)	
Occasionally	5 (23.8)	29 (17.9)	5 (29.4)	6 (20.0)	
Regularly, but less than once a month	2 (9.5)	13 (8.0)	2 (11.8)	1 (3.3)	
Regularly, at least once a week	2 (9.5)	10 (6.2)	0 (0.0)	3 (10.0)	
Regularly, at least once a week, but the lesions cease after a break from work	2 (9.5)	25 (15.4)	2 (11.8)	2 (6.7)	
11. Pruritus	10 (47.6)	62 (38.3)	8 (47.1)	12 (40.0)	0.786
12. Is the onset of the lesions sudden	6 (28.6)	54 (33.3)	4 (23.5)	7 (23.3)	0.628
13. Do symptoms occur 24–72 h after exposure?	5 (23.8)	44 (27.2)	4 (23.5)	4 (13.3)	0.453
14. Were any measures that help to restore the epidermal barrier used?	5 (23.8)	32 (19.8)	6 (35.3)	3 (10.0)	0.207
15. Any area of the body affected	10 (47.6)	75 (46.3)	8 (47.1)	10 (33.3)	0.605
16. Dryness of skin	11 (52.4)	91 (56.2)	10 (58.8)	14 (46.7)	0.781

**Table 5:** Assessment of the level of exposure to occupational skin diseases in groups differing in sex and test results.

Variable	Female N = 203	Male N = 27	*p* Test Score
OSDES (score)			
M ± SD	6.2 ± 4.7	2.7 ± 4.2	<0.001
Me [Q1;Q3]	7 [1; 10]	0 [0; 6]	
Min–Max	0–16	0–13	
OSDES-16			
Low level	71 (35.0)	20 (74.1)	<0.001
Average level	41 (20.2)	2 (7.4)	
High level	91 (44.8)	5 (18.5)	

**Table 6:** Assessment of the level of exposure to occupational skin diseases in groups with different work positions.

The Level of Exposure	Position				*p* Test Score
	Physicians N = 21 (%)	Nurses N = 162 (%)	Analytics N = 17 (%)	Others N = 30 (%)	
OSDES-16 score					0.32
M ± SD	5.0 ± 4.3	6.1 ± 4.9	6.1 ± 4.6	4.6 ± 4.4	
Me [Q1; Q3]	6 [0; 9]	6 [1; 10]	9 [1; 10]	4 [0; 9]	
Min–Max	0–13	0–16	0–12	0–12	
OSDES-16 scale rating					0.78
Low level	9 (42.9)	62 (38.3)	5 (29.4)	15 (50.0)	
Average level	5 (23.8)	30 (18.5)	3 (17.6)	5 (16.7)	
High level	7 (33.3)	70 (43.2)	9 (52.9)	10 (33.3)	

**Table 7:** Assessment of the level of exposure to occupational skin diseases in a group of patients with different work experience in the current position.

The Level of Exposure	Seniority			*p* Test Score
	<6 Lat N = 74	6–10 Lat N = 87	>10 Lat N = 69	
OSDES-16 score				0.011
M ± SD	4.5 ± 4.5	6.1 ± 4.7	6.8 ± 4.8	
Me [Q1; Q3]	3 [0; 9]	6 [1; 10]	9 [2; 11]	
Min–Max	0–14	0–16	0–15	
OSDES-16 scale rating				0.037
Low level	38 (51.4)	32 (36.8)	21 (30.4)	
Average level	15 (20.3)	17 (19.5)	11 (15.9)	
High level	21 (28.4)	38 (43.7)	37 (53.6)	

## Data Availability

Data is unavailable due to privacy restrictions.
